# Perceived Risk Perception of Future Cardiovascular Disease and Diabetes in the Postpartum Period

**DOI:** 10.3390/jpm16030137

**Published:** 2026-03-01

**Authors:** Amin Heidarikakolaki, Siew Lim, Maureen Makama, Mingling Chen, Melinda J. Hutchesson, Cheryce L. Harrison, Helen Skouteris, Helena Teede, Lisa J. Moran

**Affiliations:** 1 Monash Centre for Health Research and Implementation, Faculty of Medicine, Nursing and Health Sciences, Monash University, Clayton, Melbourne, VIC 3168, Australia; amin.heidarikakolaki@monash.edu (A.H.);; 2 Health Systems and Equity, Eastern Health Clinical School, Faculty of Medicine, Nursing and Health Sciences, Monash University, Box Hill, Melbourne, VIC 3128, Australia; siew.lim1@monash.edu; 3 Women’s, Children’s and Adolescents’ Health Program, Burnet Institute, Prahran, Melbourne, VIC 3004, Australia; 4 Department of Endocrine and Metabolic Diseases, Shanghai Institute of Endocrine and Metabolic Diseases, Ruijin Hospital, Shanghai Jiao Tong University School of Medicine, Shanghai 200025, China; 5 Shanghai National Clinical Research Centre for Metabolic Diseases, Key Laboratory for Endocrine and Metabolic Diseases of the National Health Commission of the PR China, Shanghai Key Laboratory for Endocrine Tumour, Ruijin Hospital, Shanghai Jiao Tong University School of Medicine, Shanghai 200025, China; 6 School of Health Science, College of Health Medicine and Wellbeing, University of Newcastle, Callaghan, NSW 2308, Australia; 7 Food and Nutrition Research Program, Hunter Medical Research Institute, University of Newcastle, New Lambton Heights, NSW 2305, Australia; 8 Monash Endocrine and Diabetes Units, Monash Health, Monash University, Melbourne, VIC 3168, Australia; 9 Warwick Business School, University of Warwick, Coventry CV4 7AL, UK; 10 Health and Social Care Unit, School of Public Health and Preventive Medicine, Monash University, Melbourne, VIC 3004, Australia; 11 Victorian Heart Institute, Monash Health, Monash University, Clayton, Melbourne, VIC 3168, Australia

**Keywords:** cardiovascular diseases, diabetes, postpartum, pregnancy complications, non-pregnancy conditions, risk perception, lifestyle, polycystic ovary syndrome, infertility

## Abstract

**Background/Objectives**: Risk perception of future disease may play a role in supporting lifestyle change to prevent diabetes mellitus (DM) and cardiovascular disease (CVD). It is unknown how women in the postpartum period with different cardiometabolic conditions perceive their future risk of DM and CVD, and whether this perception influences engagement in a healthy lifestyle. **Methods**: Cross-sectional study of women who delivered in the past five years (n = 497) living in Australia. Logistic regression analyses examined associations between history of pregnancy (gestational diabetes mellitus (GDM), gestational hypertension (GHP), pre-eclampsia, spontaneous preterm birth (PTB), small-for-gestational-age (SGA) infants), and non-pregnancy (polycystic ovary syndrome (PCOS), infertility) conditions with perceived risk of DM or CVD, and with lifestyle behaviours (physical activity, sedentary behaviour, and diet). **Results**: Overall, most participants had a low perceived risk of developing future DM (73.4%) and CVD (75.2%), which varied by condition type. History of GDM and GHP were associated with higher DM risk perception (OR 1.83, 95% CI 1.06, 3.15; OR 2.73, 95% CI 1.28, 5.84), whereas history of pre-eclampsia and DM were associated with higher CVD risk perception (OR 4.48, 95% CI 1.88, 10.62; OR 3.78, 95% CI 1.20, 11.88). History of PTB, SGA infant, PCOS, infertility, and lifestyle behaviours were not consistently associated with perceived risk of DM and CVD. **Conclusions**: Postpartum risk perception of developing future DM and CVD was low, even in the presence of female-specific cardiometabolic conditions. This highlights the need for greater postpartum support to enhance risk awareness and support a healthy lifestyle.

## 1. Introduction

Cardiovascular disease (CVD) is the leading cause of morbidity and mortality in women, with global healthcare expenditures expected to exceed USD $2 trillion by 2050 [1]. Diabetes mellitus (DM), a well-established risk factor for CVD, further contributes to this burden [2,3]. Women also experience additional sex-specific risk factors, including pregnancy-related cardiometabolic conditions such as gestational diabetes mellitus (GDM), gestational hypertension (GHP), pre-eclampsia, spontaneous preterm birth (PTB), intrauterine growth restriction (IUGR), and small-for-gestational-age (SGA) infants, affecting 5–30% of pregnancies [4,5]. Non-pregnancy conditions, including polycystic ovary syndrome (PCOS) and infertility, affect up to 13% and 4% of women, respectively [6,7]. These conditions differ in their clinical presentation and timing across the life course; they represent a coherent group of female-specific cardiometabolic risk factors due to their shared underlying pathophysiological mechanisms. Collectively, these conditions are associated with up to 10-fold and two-fold increased risks of type 2 DM and CVD, respectively [4,5,8,9,10,11,12,13]. These elevated risks are driven by overlapping mechanisms, including impaired hemodynamic adaptation, endothelial dysfunction, placental insufficiency, systemic inflammation, oxidative stress, insulin resistance, and altered glucose metabolism [4,5,8,9,10,11,14]. These mechanisms frequently coexist alongside modifiable risk factors, including hypertension, dyslipidaemia, obesity, sedentary behaviour, unhealthy diet, smoking, and psychosocial stress [4,5,9,14,15,16]. Substantial overlap also exists across these conditions; for example, PCOS is associated with infertility [17], and both PCOS [18] and infertility [19] are associated with a higher prevalence of cardiometabolic pregnancy complications.

The postpartum period is a crucial time during which diabetes and cardiovascular risk factors, as well as early cardiometabolic diseases, may emerge in women with these female-specific cardiometabolic conditions [20]. For women with pregnancy conditions, evidence-based statements, such as those from the American Heart Association, recommend structured follow-up within 6 weeks to 12 months postpartum to support primary prevention of CVD [21]. Recommended strategies include cardiometabolic screening and optimising nutrition, physical activity, mental and emotional well-being, and weight management [22,23,24,25,26]. Likewise, the 2023 International Evidence-Based Guideline for PCOS recommends comprehensive assessment of type 2 DM and CVD risk, alongside lifestyle optimisation [17]. While no formal postpartum guidelines currently exist for infertility, emerging evidence supports early risk stratification [27], lifestyle optimisation [28], management of shared risk factors with other conditions (including PCOS) [19], and structured follow-up, particularly following assisted reproductive technology treatment [29]. Together, these approaches underscore the importance of early identification and proactive management of cardiometabolic risk across female-specific conditions, particularly during the postpartum period.

Understanding women’s perception of cardiometabolic risk is clinically important, as risk perception is a potentially modifiable determinant of preventive health behaviour and engagement with care [30]. Risk perception influences whether individuals seek clinical follow-up, adhere to recommended screening, and engage in lifestyle interventions, particularly in preventive contexts where symptoms may be absent [30]. It is shaped by perceived susceptibility to illness, perceived severity of illness, perceived benefits of behaviour change, and perceived barriers [31], and forms the basis of several health behaviour theories, including the health belief model and the theory of fear appeals and attitude change [32,33]. In the postpartum setting, where engagement with healthcare services often declines after routine maternity care, risk perception may play a critical role in determining whether women attend recommended follow-up, undergo cardiometabolic screening, and initiate or sustain lifestyle modifications [30].

Although higher perceived CVD risk has been associated with healthier behaviours, including improved physical activity, fruit and vegetable consumption, and adequate sleep [34,35,36], findings are inconsistent [37]. Importantly, many women with female-specific cardiometabolic conditions underestimate or misperceive their risk of both type 2 DM and CVD [30,38]. Most evidence focuses on women with GDM and hypertensive disorders of pregnancy (HDP) [30], with limited research in PTB [30], IUGR [30], PCOS [38], and infertility [39]. Additionally, findings vary across conditions, with some associated with perceptions of both future type 2 DM [40] and CVD risk [41], while others are related to perception of only one of these outcomes [30]. Importantly, even when women accurately perceive themselves to be at elevated risk, this awareness alone does not consistently translate into adaptation or maintenance of healthy lifestyle behaviours in the postpartum period [30]. These findings highlight persistent clinical and research gaps in postpartum risk communication, women’s understanding of long-term cardiometabolic risk [42], and the translation of risk awareness into sustained preventive lifestyle behaviour across female-specific cardiometabolic conditions [30,43,44].

Compounding these challenges, the postpartum period is characterised by reduced healthcare follow-ups, reduced social support, and caregiving demands, which can contribute to weight retention, lower physical activity levels, and suboptimal dietary behaviours [44]. Despite growing recognition of these issues, there remains a clear gap in understanding whether perceived cardiometabolic risk among postpartum women translates into actual lifestyle intentions or behaviours, particularly across the full spectrum of female-specific conditions. Addressing this gap is clinically relevant and critical for the development of targeted interventions that not only raise awareness but also support women in taking effective prevention action. The study aimed to examine whether women with a history of female-specific conditions perceived themselves to be at greater risk of type 2 DM or CVD, and whether this risk perception was associated with planning for or engagement in lifestyle changes, compared with women without a history of these conditions.

## 2. Methods and Design

### 2.1. Study Method

This is a secondary cross-sectional analysis of a quantitative online survey conducted among women during the postpartum period [45]. The original study was designed to examine postpartum health perception and corresponding behaviours. The present analyses extend this study by specifically evaluating the association between perceived risk of type 2 DM, CVD, and lifestyle behaviours across a broader range of female-specific cardiometabolic conditions. This addresses a distinct research question that was not the primary focus of the original study and provides additional insight into the translation of risk perception into preventive health behaviours in the postpartum period.

The study was conducted according to the guidelines of the Declaration of Helsinki and approved by Monash University Human research ethics committee (HREC) (approved on 21 September 2021, project number 29273). Informed consent of all participants was provided, and detailed methods were previously described elsewhere [45].

### 2.2. Participants

Participants were recruited throughout Australia via an external cross-panel market research provider (Octopus Group) between 9 and 22 November 2021 [45]. Eligible participants were ≥18 years, had experienced pregnancy and delivered their baby in the last 5 years, were not currently pregnant, and were living with their child in Australia. The postpartum period was defined as extending up to five years after delivery to increase the sample size and capture variation in both short and long-term cardiometabolic risk factors and health behaviours [46], corresponding to the first 2000 days as a critical window for shaping women’s health [47]. In this specific sub-study, women who were experiencing or had experienced menopause were also excluded to minimise heterogeneity in hormonal status and focus on a reproductive-aged population. Participants were broadly representative of the Australian population in terms of geographic distribution and residential location, as defined by the Australian Bureau of Statistics [45,48].

### 2.3. Data Collection

A questionnaire was developed by the research team, pilot tested in postpartum women, incorporating items from established instruments validated, in general, adult populations, and used in related studies [45,49]. It was self-administered 20–30 min duration [45], comprising multiple-choice, open-ended, and Likert-type scale questions.

#### 2.3.1. Demographics

For this study, the self-assessed questions were analysed across the following domains: demographics [age (years), body mass index (BMI) (kg/m^2^), number of children < 18 living in house, number of adults ≥ 18 years living in the house, age of youngest child (years), ethnicity (three sub-categories), country of birth (two categories), marital status (three categories), education level (four categories), employment (three categories) and income (four categories)].

#### 2.3.2. Medical Conditions

The history of conditions was assessed based on self-reported history of diagnoses or treatment, which may be subject to recall bias, miscalculation, or overlap between condition groups (such as PCOS and infertility). Participants were asked, ‘Have you ever had, been diagnosed with, or been treated for diabetes (excluding gestational diabetes), GDM (diabetes onset in pregnancy), GHP (high blood pressure that started during pregnancy), pre-eclampsia, PTB (delivery before 37 weeks of pregnancy), delivery of a baby with a birth weight less than 2500 g after 37 weeks of pregnancy (SGA infant), PCOS, or infertility?’ For analyses, these conditions were grouped into the following categories: diagnosed with history of pregnancy cardiometabolic conditions (GDM, GHP, pre-eclampsia, PTB, SGA infant), history of non-pregnancy conditions (diabetes, PCOS, and infertility), and history of more than one condition (GDM, GHP, pre-eclampsia, PTB, SGA infant, DM, PCOS, and infertility).

#### 2.3.3. Risk Perception

Risk perception of type 2 DM or CVD was assessed by a brief four-item self-reported questionnaire adapted from the Risk Perception Survey for Developing Diabetes (RPS-DD) [50], which has been previously used in research with women with a history of GDM [51] and HDP [52]. Adaptation of the RPS-DD for CVD involved substituting references to diabetes with CVD and adding clarifying examples (e.g., heart disease, hypertension, or stroke) to enhance relevance and comprehension for this study and for CVD. This adaptation is conceptually appropriate as the instrument assesses perceived susceptibility and behavioural response, constructs shared across cardiometabolic conditions with overlapping risk pathways and prevention strategies, although it may not capture all CVD-specific dimensions of risk perception.

Specifically, risk perception was assessed using the following items: (1) ‘What do you think your risk of developing diabetes is over the next 10 years?’; (2) ‘If you don’t change your lifestyle behaviours, such as diet or exercise, what is your risk or chance of getting diabetes over the next 10 years?’; (3) ‘Have you recently made changes in any lifestyle behaviours that you believe will lower your chance of getting diabetes?’; (4) ‘Are you planning to make changes in any lifestyle behaviours in the near future that you believe will lower your chances of getting diabetes?’ (with responses of “yes” or “no”). Items 1 and 2 were converted into two categories of “low risk” (“almost no risk” and “slight risk”) and “high risk” (“moderate risk” and “high risk”) to improve interpretability, reduce sparse response categories, and maintain consistency with prior applications of the RPS-DD. This approach reflects a meaningful distinction between lower and elevated perceived risk and is suitable for examining associations with lifestyle intentions and behavioural change in epidemiological analyses [53].

#### 2.3.4. Lifestyle Behaviours

Self-reported physical activity was assessed using items from Active Australia’s 1999 National Physical Activity Survey [54,55], which asked, ‘State how many times you did each type of activity last week (only activities ≥ 10 min)’ across activities, including walking for recreation or exercise, moderate or vigorous intensity for leisure time, and vigorous intensity for household or gardening. Total physical activity was calculated as metabolic equivalent minutes per week (MET.min/week) using standard scoring procedures. Sitting time was assessed by self-reported time spent sitting on weekdays and weekends, with average daily sitting time calculated accordingly. For analyses, these items were treated as continuous data and reported as: total physical activity (minutes/week), total brisk walking (minutes/week), total moderate activity (minutes/week), total vigorous activities (minutes/week), and total sitting time (hours/day).

Self-reported dietary intake was assessed using a diet short-screener based on the Australian Dietary Guidelines (ADG) and Australian Guide to Healthy Eating (AGHE) [56,57]. Participants reported the estimated number of serves consumed daily or weekly from each food group (grains, vegetables and legumes, fruit, dairy or alternatives, and meat products and equivalents, and extras/discretionary choices), accounting for all foods eaten, including ingredients in recipes, mixed meals, take-away foods, and restaurant dishes. Standard serve sizes were provided from the ADG and AGHE, and each food group was illustrated with images to assist in portion size estimation. For analyses, the estimated number of serves eaten from each food group per day was converted to continuous data and reported as serves of grain, vegetable, fruit, dairy products and equivalents, meat products and equivalents, and extras/discretionary foods as serves/day.

### 2.4. Statistical Analyses

All analyses were performed using Stata 19.5 (Stata Corp LLC, College Station, TX, USA) [58]. Statistical significance was set at *p* < 0.05. Descriptive statistics were used to summarise participants’ characteristics, distribution profiles, and perceived risk of future type 2 DM and CVD. Categorical variables were presented as frequencies and percentages, while continuous variables were reported as means and standard deviations, as all continuous variables were normally distributed. Group differences in participant characteristics, distribution modes, preference profiles, and risk perception were assessed using independent sample *t*-tests for normally distributed continuous variables (e.g., age, BMI) to compare group means, and Mann–Whitney U tests, or Pearson’s chi-square tests, as appropriate.

Associations between pregnancy conditions or non-pregnancy conditions (individually or in groups) and perceived risk of either type 2 DM or CVD were examined using univariable and multivariable logistic regression. Two models were fitted: Model 1 was crude (unadjusted), and Model 2 was adjusted for age, BMI, ethnicity, education level, income, employment status, marital status, number of children in the household, and postpartum age. The choice of these variables was based on established literature [45,49], significance and hypothesis testing. In addition, associations between type 2 DM or CVD risk perception and lifestyle behaviours were explored using univariable and multivariable logistic regression models, including an unadjusted model (Model 1) and an adjusted model (Model 2) with the same covariates as described above. Linearity assumptions for continuous lifestyle behaviour variables were evaluated using graphical methods and were considered reasonable.

Subgroup analyses were also conducted to examine whether associations between lifestyle behaviours and perceived risk of either type 2 DM or CVD differed by condition status, by including interaction terms between risk perception and individual or grouped conditions. Subgroup analyses were restricted to clinically relevant binary or small-category splits to ensure adequate sample size and stable estimates within each stratum. These analyses aimed to determine whether the association between lifestyle behaviours and risk perception varied significantly across different categories of the conditions, as indicated by differences in the regression coefficients (B). These analyses were also fitted using two models: Model 1 (crude) and Model 2 (adjusted for the covariates as mentioned above). Results were interpreted cautiously, emphasising effect sizes and confidence intervals rather than statistical significance alone.

## 3. Results

### 3.1. Participants’ Characteristics

A total of 497 pre-menopausal women who reported a perceived risk of either type 2 DM or CVD were included in the present analysis. Participant characteristics of the population are reported in Table 1 and Table S1. The mean age was 33.6 ± 5.5 years, and BMI was 27.2 ± 6.9 kg/m^2^. On average, participants reported having 2.0 ± 1.1 children under the age of 18 years, and the age of the youngest child was 4.0 ± 1.9 years. More than half of participants (54.5%) were born in Australia, the majority were married (88.1%), and educational level, annual household income, and employment status were diverse across the population with similar distributions. Ethnically, 51.7% identified as Oceanian, 34.2% as Asian, and 12.3% as belonging to other ethnic backgrounds.

Overall, 40.0% reported a history of pregnancy conditions, 14.7% had a history of non-pregnancy conditions, and 15.5% had more than one condition. Specifically, the prevalence of conditions was DM (3.4%), GDM (19.2%), GHP (7.7%), pre-eclampsia (6.6%), PTB (12.5%), SGA infant (4.6%), PCOS (8.7%), and infertility (5.8%). Higher BMI was significantly related to an increased risk of pregnancy conditions (*p* = 0.002), non-pregnancy conditions (*p* = 0.004), and more than one condition (*p* < 0.001). Having more children under 18 years increased the risk of both pregnancy-related conditions (*p* = 0.007) and more than one condition (*p* < 0.001). The age of the youngest child was associated with an increased risk of both pregnancy-related (*p* = 0.049) and non-pregnancy conditions (*p* = 0.015). Being born in Australia (*p* < 0.001) and Asian ethnicity (*p* = 0.010) were also associated with an increased risk of non-pregnancy conditions.

### 3.2. Risk Perception (Primary Outcome)

#### 3.2.1. Risk Perception Characteristics

**Figure 1:**
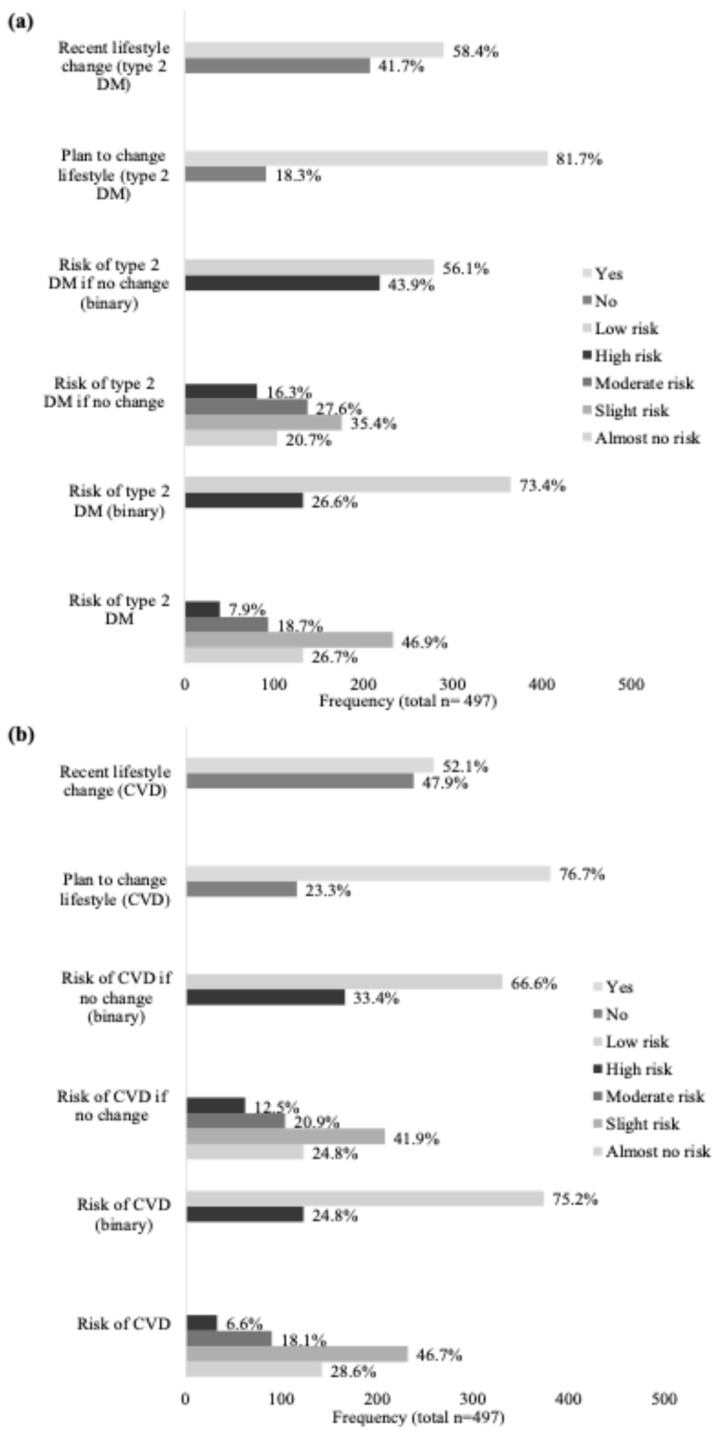
Risk perception of type 2 DM (**a**) and CVD (**b**) for the total population.

The risk perception characteristics of the population are reported in Figure 1. Most participants considered their risk of developing type 2 DM (73.4%) and CVD (75.2%) to be low. Even if no lifestyle changes were made, 56.1% and 66.6% still perceived their risk as low for type 2 DM and CVD, respectively. Plans to adopt lifestyle changes were reported by 81.7% of participants for type 2 DM and 76.7% for CVD, while 58.4% and 52.1%, respectively, had recently made changes to reduce their risk.

#### 3.2.2. Perceived Risk of Type 2 DM by Each Condition

The association between pregnancy conditions or non-pregnancy conditions and perceived risk of type 2 DM is provided in Table 2. Women with a history of GDM were more likely to perceive a higher risk of developing type 2 DM (OR 1.83, 95% CI 1.06, 3.15), including in the absence of lifestyle change (OR 1.78, 95% CI 1.06, 2.99), and were more likely to be planning (OR 4.66, 95% CI 1.61, 13.50) or to have recently made lifestyle changes (OR 1.83, 95% CI 1.07, 3.13). Similarly, those with a history of GHP were more likely to perceive a higher risk of developing type 2 DM (OR 2.73, 95% CI 1.28, 5.84), a higher risk if no lifestyle changes were made (OR 2.63, 95% CI 1.19, 5.79), and a recent lifestyle change (OR 3.09, 95% CI 1.26, 7.56). Women with a history of pre-eclampsia were more likely to perceive type 2 DM risk only in the absence of lifestyle change (OR 2.61, 95% CI 1.06, 6.43). Women with any pregnancy condition were more likely to perceive type 2 DM risk (OR 2.01, 95% CI 1.25, 3.21), including if no lifestyle changes were made (OR 1.69, 95% CI 1.11, 2.57), and women with multiple conditions also reported higher perceived risk in this scenario (OR 1.92, 95% CI 1.05, 3.52).

#### 3.2.3. Perceived Risk of CVD by Each Condition

The association between pregnancy or non-pregnancy conditions and perceived risk of CVD is provided in Table 3. Women with a history of DM were more likely to perceive themselves at risk of CVD (OR 3.78, 95% CI 1.20, 11.88), including in the absence of lifestyle modifications (OR 3.76, 95% CI 1.15, 12.31), and to have recently made lifestyle changes to reduce risk (OR 3.99, 95% CI 1.02, 15.60). For planning lifestyle change, all women with DM reported such an intention, resulting in perfect prediction and exclusion of this variable from the regression model. Additionally, women with a history of pre-eclampsia were more likely to perceive a higher risk of CVD (OR 4.48, 95% CI 1.88, 10.62) and a higher perceived risk in the absence of lifestyle change (OR 4.37, 95% CI 1.78, 10.61). Women with a history of GHP perceived higher CVD risk only in unadjusted analyses (OR 2.39, 95% CI 1.21, 4.72), while in adjusted analyses, they were more likely to report recent lifestyle changes to reduce CVD risk (OR 2.86, 95% CI 1.26, 6.49).

##### Key Summary Results of Risk Perception Between Type 2 DM, CVD, and Each Condition

Overall, while most participants perceived themselves at low risk of developing type 2 DM or CVD even if no lifestyle changes were made, a substantial proportion reported planning or recently making lifestyle modifications to reduce risk. Women with a history of pregnancy-related conditions, particularly GDM, GHP, or pre-eclampsia, were more likely to perceive a higher risk of type 2 DM and, to a lesser extent, CVD, and were also more likely to report recent or planned lifestyle changes.

### 3.3. Lifestyle Behaviours and Risk Perception (Exploratory Outcome)

#### 3.3.1. Lifestyle Behaviours and Perceived Risk of Type 2 DM or CVD

The associations between lifestyle behaviours and perceived risk of type 2 DM or CVD are provided in Table 4. Those who reported a higher perceived risk of type 2 DM also reported greater daily intakes of meat (coefficient 0.26 serves/day, 95% CI 0.01, 0.50) and milk (coefficient 0.25 serves/day, 95% CI 0.01, 0.48). Furthermore, those with a higher perceived risk of CVD reported greater daily intake of grains (coefficient 0.41 serves/day, 95% CI 0.07, 0.74). Overall, effect sizes were small, indicating modest differences in self-reported behaviours according to perceived risk.

#### 3.3.2. Subgroup Analyses

##### Type 2 DM Risk Perception and Lifestyle Behaviours by Each Condition

Subgroup analyses of type 2 DM risk perception and lifestyle behaviours by history of individual and grouped conditions are shown in Tables S2, S3 and S6. In adjusted analyses, women with SGA infants who reported higher type 2 DM risk perception also reported greater sitting time (coefficient 8.44 h/day, 95% CI 1.85, 15.03). Women with PCOS also reported less brisk walking (coefficient −132.4 min/week, 95% CI −252.76, −12.03). Among women with multiple conditions, those who reported higher type 2 DM risk perception also reported more sitting time (coefficient 2.88 h/day, 95% CI 0.21, 5.54).

##### CVD Risk Perception and Lifestyle Behaviours by Each Condition

Subgroup analyses of CVD risk perception and lifestyle behaviours by history of individual and grouped conditions are shown in Tables S4, S5 and S7. In adjusted analyses, women with PE who reported higher CVD risk perception also reported greater sitting time (coefficient 3.54 h/day, 95% CI 0.08, 7.00) and lower vegetable intake (coefficient −1.20 serves/day, 95% CI −2.12, −0.28). Women with GHP who reported higher CVD risk perception also reported greater moderate physical activity (coefficient 135.94 min/week, 95% CI 18.75, 253.13), sitting time (coefficient 4.80 h/day, 95% CI 1.65, 7.95), and fruit intake (coefficient 0.94 serves/day, 95% CI 0.17, 1.71). In the subgroup of women with pregnancy conditions, those who reported higher CVD risk perception also reported lower vegetable consumption (coefficient −0.86 serves/day, 95% CI −1.53, −0.19). In the subgroup of women with multiple conditions, those who reported higher CVD risk perception also reported greater sitting time (coefficient 3.29 h/day, 95% CI 0.82, 5.76).

##### Key Summary Result of Exploratory Outcomes

Overall, perceived risk showed only modest associations with lifestyle behaviours, with some condition-specific variations observed. These analyses were exploratory and undertaken to assess potential associations and are presented to inform future research.

## 4. Discussion

This study reported that the perceived risk of type 2 DM and CVD was generally low in postpartum women and differed according to specific female-specific cardiometabolic conditions and characteristics such as BMI, number of children in the house, age of youngest child, and ethnicity. In adjusted analyses, women experiencing HDPs, including GHP or pre-eclampsia, reported a higher perceived risk of both type 2 DM and CVD. Experiencing DM was additionally associated with an increased perceived risk of CVD, whereas experiencing GDM was only associated with increased perceived risk of future type 2 DM. Experiencing other conditions, including PTB, SGA infant, PCOS, and infertility, were not associated with perceived risk. In general, increased perceived risk of type 2 DM or CVD was not consistently associated with changes in lifestyle behaviours.

A history of DM was associated with increased perceived CVD risk, and all women with DM reported planning or recently initiating lifestyle changes to reduce this risk. This aligns partially with the current limited but mixed literature reporting that women with DM have low–moderate CVD risk perception [59]. The observed higher risk perception in our cohort may be influenced by national strategies [60] and targeted public health campaigns, such as “Take Diabetes 2Heart” [61], “REDFEB” [62], as well as international campaigns [63,64,65]. It may also reflect women’s awareness of type 2 DM-related risk factors, including high BMI [66], excessive weight gain [67], and high cholesterol [52], which are strongly linked with CVD.

A history of GHP or pre-eclampsia was also associated with a higher perceived risk for future type 2 DM or CVD. This contrasts with earlier studies reporting limited awareness of these risks [68,69,70]. For instance, previous studies reported that only 14.4 and 4.7% of women with pre-eclampsia perceived an increased risk of future type 2 DM2 and CVD, respectively [68], while another study reported that only 20% demonstrated good knowledge of CVD [70]. Conversely, 68% of women with HDP reported being unaware of their increased CVD risk [69]. These differences may be related to participant characteristics, including the older age of the youngest child in our sample (mean 4 years versus 1 year in Mpalatsouka et al. and Kassab et al. [68,70]), and higher education levels compared with previous studies [70]. Differences in survey methods may also contribute; our study used direct questions, whereas Slater et al. employed indirect approaches from biological markers such as obstetric history, blood pressure, lipids, or glucose [69]. Additionally, a temporal shift in risk awareness due to clinical guideline publications [71] and increasing public campaigns [61,62,72] may further explain our findings. Despite this, mixed population-level awareness of type 2 DM and CVD remains among both women and some clinicians [69,73], which may limit consistent counselling and follow-up care [74]. Further research is warranted to investigate these concepts across large population-based longitudinal studies using standardised measurement tools.

As previously reported [40,75], a history of GDM was associated with increased perceived risk of type 2 DM, likely due to regular contact with health professionals, such as endocrinologists, during or after pregnancy. Novel to our study, we reported that a history of GDM was not associated with perceived CVD risk, despite robust evidence reporting that women with prior GDM have up to twofold higher risk of future CVD [76]. This under-recognition may be associated with women receiving more frequent counselling on GDM-type 2 DM links [76], compared to GDM-CVD links [30,41,77,78], and perceiving CVD as a condition confined to pregnancy by both women and health professionals during postpartum follow-up [79]. Limited health literacy regarding CVD and inconsistent messaging across healthcare settings may further contribute to this gap [80]. These findings highlight the importance of postpartum education and support addressing both type 2 DM and CVD risk in higher-risk women during postpartum.

We observed that a history of PTB, SGA infant, PCOS, and infertility were not associated with perceived risk of any conditions. This highlight a novel finding, as these conditions are associated with up to 10-fold and two-fold increased risk of type 2 DM and CVD, respectively [4,5,9,10,12,13,14,39,81], yet remain under-recognised risk factors by women. Low risk perception in this context has important implications, as it may delay engagement with preventive care, reduce motivation for lifestyle modification, and limit opportunities for early intervention during key reproductive and postpartum windows [30,82,83]. Despite shared cardiometabolic mechanisms with type 2 DM, GDM and HDP, such as insulin resistance, chronic inflammation, endothelial dysfunction, and the higher burden of pregnancy conditions such as GDM and pre-eclampsia in women with infertility and PCOS [4,16,18,38,81,84], these links remain poorly recognised [85]. In practice, reproductive-aged women attending postpartum follow-ups or fertility clinics could extend their services to assist women for early risk stratification, counselling, and referrals to preventive interventions. This is particularly relevant in the postpartum period, where barriers to sustaining healthy lifestyle change, such as competing childcare demands and limited social support, are well recognised [45]. Strengthening awareness and communication of cardiometabolic risk across patients, practitioners, and health systems may therefore be essential to translating the growing recognition of these conditions as risk markers into meaningful prevention strategies [17,30,77,86,87]. Further research is needed to improve awareness and communication of future risk across patients, practitioners, and health systems.

The relationship between risk perception and lifestyle behaviours is complex. Some studies suggest that higher perceived risk of type 2 DM or CVD may motivate preventive behaviours such as improving dietary and physical activity [34,88,89]. However, the association between perceived risk and actual sustained lifestyle change is far less consistent, with several studies reporting weak or no associations [90,91,92,93]. This discrepancy may reflect the influence of multiple intervening factors that constrain women’s capacity to act on risk awareness during postpartum. Psychological factors, including depression, stress, competing life priorities, and reduced self-efficacy, may limit behavioural responses even in the presence of heightened perceived risk [94,95]. In postpartum, caregiving demands, fatigue, and time constraints may further reduce opportunities for engaging in healthy lifestyle behaviours [96]. In addition, limited health literacy may impede understanding of recommended lifestyle changes or their relevance, while healthcare system barriers, including fragmented follow-ups, limited access to tailored preventive services, and insufficient counselling, may impede sustained behaviour change [44]. However, we interpret these findings as exploratory given heterogeneity in factors including study designs, outcome measures, and follow-up duration. Overall, the available evidence suggests that awareness of type 2 DM or CVD risk alone is insufficient to reliably drive behavioural change.

In the postpartum period, a recent systematic review reported that although women with GDM, HDP, and IUGR perceive a modest risk of future type 2 DM and CVD and report motivation to adopt preventive lifestyle behaviours, such as regular physical activity, healthy diet, and weight management, this awareness rarely translates into sustained lifestyle modification [30]. Specifically, while women reported higher odds of intentions to change lifestyle or having recently implemented preventive behaviours for type 2 DM (GDM or GHP) or CVD (DM or GHP) [41,92,97,98,99,100], inconsistent improvements in measured lifestyle behaviours were observed. Several studies reported low adherence or unsustained changes to healthy lifestyle behaviours [51,100,101], and only inconsistent associations between risk perception and actual lifestyle behaviour changes observed [92,93]. This suggests a gap between risk perception and effective behavioural action.

While raising awareness and improving risk perception of chronic disease is critical, excessive personal responsibility is often placed on women, particularly during the postpartum period, which is a time of significant physical and emotional demands [30,44]. In addition, suboptimal communication from healthcare professionals may further undermine risk understanding and engagement in preventive care [30]. This underscores the need for greater support being needed from medical and allied healthcare practitioners in helping women understand the long-term risks of female-specific type 2 DM and CVD risk factors, and in achieving appropriate screening and lifestyle behaviours. Our findings highlight the need for structured antenatal and postpartum care models that actively engage women with female-specific cardiometabolic conditions, incorporating risk assessment, tailored lifestyle counselling, screening, and long-term follow-up. This can aid in translating awareness and intentions into meaningful and sustained engagement with primary prevention behaviours, while addressing barriers such as fatigue, caregiving demands, family commitments, and insufficient practical guidance [42,82].

A key strength of this study is the comprehensive assessment of perceived risk for both type 2 DM and CVD in postpartum women across a broad range of female-specific cardiometabolic conditions. The use of a large, nationally representative sample capturing social and cultural diversity further supports the potential generalisability of the findings. In addition, the use of validated risk perception measures also strengthens the methodological rigour and practical relevance of the study. Several limitations are also acknowledged, including the self-reported assessment, which may be less accurate or sensitive compared with more comprehensive measurement tools, such as 24 h dietary recalls or weighed food diaries for diet, and objective wearable tools, including accelerometers, for physical activity. Another limitation is that the survey question assessed perceived risk of DM overall rather than distinguishing between type 1 and type 2 DM, which may have introduced misclassification and limited the specificity of the findings. Additionally, the cross-sectional design precludes casual inference, preventive conclusions about the directionality of associations between risk perception and lifestyle behaviour or their sustainability over time. Finally, the population was nationally representative and lived in Australia; the findings may not be fully generalisable to populations outside the Australian postpartum context or to women with different access levels to healthcare services. Despite these limitations, the consistency of the results with existing literature reinforces the reliability and applicability of the findings.

## 5. Conclusions

Findings emphasise the need for a more proactive and structured approach to cardiometabolic risk management during the postpartum period. Inconsistent or low perceived risk of type 2 DM and CVD across female-specific risk factors suggests missed opportunities for early prevention and engagement in lifestyle behaviours. Embedding routing risk assessment within postpartum care, alongside stronger support from health professionals, may help translate risk awareness into meaningful engagement with screening and lifestyle interventions. This is particularly relevant for those with a history of GHP, pre-eclampsia, and DM for CVD risk perception, and for those with a history of GDM for perceived risk of type 2 DM. These findings highlight gaps in understanding the link between female-specific conditions and future type 2 DM and CVD, particularly for those women experiencing reproductive conditions such as PCOS and infertility. Importantly, awareness of future type 2 DM and CVD risk alone appears insufficient to motivate lifestyle behaviour changes. Future research should focus on enhancing structured follow-up by integrating risk assessment and communication within routine postpartum care, to optimise understanding and facilitate sustained preventive screening and healthy lifestyle behaviours, particularly for those with a history of cardiometabolic conditions.

## Figures and Tables

**Table 1:** Participant characteristics.

Variable		Number/Mean (SD/%) n Total = 497	Any Pregnancy Conditions n = 199 (40%)	Any Non-Pregnancy Conditions n = 73 (14.7%)	More than One Condition n = 77 (15.5%)	Overall *p*-Value		
						Preg-Con	Non-Preg-Con	More-Than
**Age (years)**		33.6 (5.5)	33.9 (5.9)	35.1 (5.2)	34.4 (5.9)	0.357	0.015	0.210
**BMI (kg/m^2^)**		27.2 (6.9)	28.4 (7.2)	29.4 (8.4)	30.5 (8.3)	0.002	0.004	0.000
**Number of children in house (<18 years old)**		2 (1.1)	2.2 (1.2)	2.2 (1.2)	2.5 (1.5)	0.007	0.171	0.000
**Number of adults in house (≥18 years old)**		1.4 (0.84)	1.4 (0.81)	1.21 (0.71)	1.29 (0.78)	0.942	0.088	0.397
**Age of youngest child**		4 (1.9)	3.8 (1.8)	4.5 (1.9)	3.9 (1.8)	0.049	0.015	0.510
**Country of birth**	Australia	271(54.5)	117 (58.8)	54 (74)	49 (63.6)	0.119	0.000	0.081
	Overseas	226 (45.5)	82 (41.2)	19 (26)	28 (36.4)			
**Marital status**	Never married	30 (6.0)	17 (8.5)	4 (5.5)	7 (9.1)	0.102	0.524	0.274
	Married/De facto	438 (88.1)	169 (84.9)	63 (86.3)	64 (83.1)			
	Separated/Divorced	27 (5.4)	13 (6.5)	6 (8.2)	6 (7.8)			
**Education level**	Secondary/high school	121 (24.4)	54 (27.1)	21 (28.8)	23 (29.9)	0.112	0.500	0.083
	Diploma/advanced diploma	97 (19.5)	41 (20.6)	16 (21.9)	18 (23.4)			
	University degree	148 (29.8)	64 (32.2)	22 (30.1)	25 (32.5)			
	Graduate/postgraduate degree	128 (25.8)	40 (20.1)	14 (19.2)	11 (14.3)			
**Ethnicity**	Oceanian	257 (51.7)	111 (56.6)	49 (69.0)	49 (65.3)	0.350	0.010	0.052
	Asian	170 (34.2)	62 (31.6)	15 (21.1)	18 (24.0)			
	Other	61 (12.3)	23 (11.7)	7 (9.9)	8 (10.7)			
**Income ($AUD)**	$0–$49,999	76 (15.3)	32 (17.4)	12 (17.1)	17 (23.0)	0.673	0.864	0.198
	$50,000–$99,999	149 (30.0)	59 (32.1)	25 (35.7)	24 (32.4)			
	$100,000–$149,999	148 (29.8)	53 (28.8)	20 (28.6)	17 (23.0)			
	>$150,000	93 (18.7)	40 (21.7)	13 (18.6)	16 (21.6)			
**Employment**	Homemaker/Student/Government assistance	154 (31.0)	70 (35.7)	23 (31.5)	32 (42.1)	0.137	0.997	0.070
	Full-time employment	149 (30.0)	51 (26.0)	22 (30.1)	17 (22.4)			
	Part-time/Casual employment	186 (37.4)	75 (38.3)	28 (38.4)	27 (35.5)			

Data were analysed according to the type of question asked: normally distributed continuous variables (age, BMI) were compared using independent sample *t*-tests; non-normally distributed continuous variables were compared using Mann–Whitney U tests; categorical variables (ethnicity, marital status, education level, employment, conditions) were compared using Pearson’s chi-square tests as appropriate. SD, standard deviation; BMI, body mass index; n, number; Any preg-con, any pregnancy conditions (including GDM, GHP, pre-eclampsia, PTB, SGA infant); Any non-preg-con, any non-pregnancy conditions (including DM, PCOS, infertility); more-than, more than one condition (including GDM, GHP, pre-eclampsia, PTB, SGA infant, DM, PCOS, infertility); $AUD, Australian dollar.

**Table 2:** The association between type 2 diabetes mellitus risk perception and cardiometabolic conditions.

Condition	Type 2 DM Risk Perception OR (95% CI)		Type 2 DM Risk If No Lifestyle Changes OR (95% CI)		Type 2 DM Risk and Plans to Change Lifestyle OR (95% CI)		Type 2DM Risk and Recent Lifestyle Changes OR (95% CI)	
	Unadjusted	Adjusted *	Unadjusted	Adjusted *	Unadjusted	Adjusted *	Unadjusted	Adjusted *
**GDM**	2.20 (1.36, 3.58)	**1.83** **(1.06, 3.15)**	2.22 (1.40, 3.53)	**1.78** **(1.06, 2.99)**	5.99 (2.14, 16.79)	**4.66** **(1.61, 13.50)**	2.00 (1.22, 3.27)	**1.83** **(1.07, 3.13)**
**GHP**	2.96 (1.51, 5.81)	**2.73** **(1.28, 5.84)**	3.71 (1.79, 7.67)	**2.63** **(1.19, 5.79)**	4.35 (1.03, 18.41)	4.01 (0.86, 18.62)	3.02 (1.35, 6.72)	**3.09** **(1.26, 7.56)**
**Pre-eclampsia**	1.97 (0.93, 4.19)	1.93 (0.77, 4.80)	2.64 (1.24, 5.65)	**2.61** **(1.06, 6.43)**	0.93 (0.37, 2.34)	0.85 (0.30, 2.45)	0.78 (0.38, 1.62)	0.90 (0.38, 2.14)
**PTB**	1.54 (0.86, 2.74)	1.43 (0.75, 2.72)	1.02 (0.59, 1.75)	0.90 (0.49, 1.66)	0.72 (0.38, 1.38)	0.66 (0.33, 1.35)	0.86 (0.50, 1.48)	0.87 (0.48, 1.57)
**SGA infant**	0.69 (0.23, 2.09)	0.37 (0.08, 1.80)	1.25 (0.52, 3.01)	1.11 (0.37, 3.28)	0.95 (0.31, 2.90)	0.61 (0.16, 2.32)	0.99 (0.41, 2.40)	0.88 (0.30, 2.62)
**PCOS**	2.04 (1.05, 3.96)	1.61 (0.76, 3.43)	1.65 (0.87, 3.14)	1.56 (0.75, 3.23)	1.10 (0.47, 2.57)	1.41 (0.52, 3.77)	1.32 (0.68, 2.57)	1.30 (0.62, 2.73)
**Infertility**	1.20 (0.51, 2.80)	0.85 (0.32, 2.29)	1.89 (0.87, 4.09)	1.46 (0.60, 3.57)	1.0 (0.38, 2.80)	1.17 (0.39, 3.53)	0.73 (0.34, 1.57)	0.93 (0.39, 2.23)
**Any pregnancy condition**	2.23 (1.47, 3.38)	**2.01** **(1.25, 3.21)**	2.01 (1.38, 2.92)	**1.69** **(1.11, 2.57)**	1.92 (1.15, 3.20)	1.62 (0.91, 2.88)	1.40 (0.96, 2.03)	1.44 (0.94, 2.19)
**Any non-pregnancy condition**	1.62 (0.89, 2.94)	1.29 (0.65, 2.56)	1.42 (0.81, 2.49)	1.21 (0.64, 2.30)	0.91 (0.45, 1.83)	1.10 (0.49, 2.48)	0.99 (0.57, 1.74)	1.05 (0.56, 1.99)
**More than one condition**	1.87 (1.08, 3.24)	1.49 (0.79, 2.79)	2.75 (1.60, 4.71)	**1.92** **(1.05, 3.52)**	1.74 (0.80, 3.78)	1.42 (0.60, 3.34)	1.47 (0.85, 2.52)	1.45 (0.78, 2.69)

Data were analysed using univariable and multivariable logistic regression. * Adjusted for age, BMI, ethnicity, education, income, employment status, marital status, children in the household, age of youngest child. OR, odds ratio; 95% CI, 95% confidence interval; BMI, body mass index; GDM, gestational diabetes mellitus; GHP, gestational hypertension; PTB, spontaneous preterm birth; SGA infant, small for gestational age; PCOS, polycystic ovary syndrome; Any pregnancy conditions (including GDM, GHP, pre-eclampsia, PTB, SGA infant); Any non-pregnancy conditions (including PCOS, infertility); More than one condition (including GDM, GHP, pre-eclampsia, PTB, SGA infant, PCOS, infertility); DM, diabetes mellitus.

**Table 3:** The association between cardiovascular disease risk perception and cardiometabolic conditions.

Condition	CVD Risk Perception OR (95% CI)		CVD Risk If No Lifestyle Changes OR (95% CI)		CVD Risk and Plans to Change Lifestyle OR (95% CI)		CVD Risk and Recent Lifestyle Changes OR (95% CI)	
	Unadjusted	Adjusted *	Unadjusted	Adjusted *	Unadjusted	Adjusted *	Unadjusted	Adjusted *
**GDM**	0.90 (0.54, 1.51)	0.77 (0.42, 1.38)	1.11 (0.70, 1.77)	0.89 (0.53, 1.50)	1.16 (0.68, 1.99)	0.88 (0.49, 1.59)	0.88 (0.56, 1.36)	0.71 (0.43, 1.16)
**GHP**	**2.39** **(1.21, 4.72)**	2.13 (0.98, 4.65)	1.89 (0.97, 3.68)	1.75 (0.83, 3.69)	1.38 (0.59, 3.22)	1.27 (0.51, 3.20)	2.76 (1.31, 5.82)	**2.86** **(1.26, 6.49)**
**Pre-eclampsia**	3.59 (1.75, 7.34)	**4.48** **(1.88, 10.62)**	2.93 (1.43, 5.99)	**4.37** **(1.78, 10.61)**	0.68 (0.31, 1.48)	0.67 (0.27, 1.64)	0.97 (0.48, 1.98)	1.17 (0.50, 2.70)
**PTB**	1.29 (0.71, 2.32)	1.19 (0.62, 2.29)	0.94 (0.53, 1.66)	0.87 (0.47, 1.64)	1.05 (0.56, 1.98)	1.22 (0.61, 2.44)	1.05 (0.62, 1.79)	1.22 (0.68, 2.19)
**SGA infant**	1.08 (0.41, 2.80)	1.17 (0.38, 3.58)	0.69 (0.27, 1.79)	0.82 (0.27, 2.51)	1.10 (0.40, 3.03)	1.47 (0.43, 4.94)	1.20 (0.52, 2.80)	1.14 (0.42, 3.13)
**PCOS**	1.36 (0.68, 2.69)	0.98 (0.45, 2.12)	0.96 (0.49, 1.87)	0.74 (0.35, 1.54)	0.88 (0.43, 1.80)	1.45 (0.55, 3.84)	1.06 (0.57, 1.99)	1.26 (0.63, 2.54)
**Infertility**	1.17 (0.50, 2.71)	0.70 (0.26, 1.90)	0.89 (0.40, 2.00)	0.57 (0.22, 1.47)	1.49 (0.56, 4.00)	1.51 (0.53, 4.33)	0.73 (0.34, 1.55)	0.65 (0.27, 1.51)
DM *	4.64 (1.73, 12.41)	3.78 **(1.20, 11.88)**	3.84 (1.40, 10.51)	3.76 **(1.15, 12.31)**	- **	- **	4.48 (1.27, 15.78)	3.99 **(1.02, 15.60)**
**Any pregnancy condition**	1.38 (0.90, 2.12)	1.32 (0.81, 2.15)	1.19 (0.80, 1.75)	1.08 (0.69, 1.69)	1.02 (0.67, 1.57)	0.93 (0.57, 1.52)	0.97 (0.67, 1.40)	0.91 (0.60, 1.38)
**Any non-pregnancy condition**	1.49 (0.87, 2.56)	1.05 (0.56, 1.95)	1.12 (0.67, 1.89)	0.89 (0.49, 1.61)	1.21 (0.66, 2.23)	1.29 (0.65, 2.55)	1.29 (0.78, 2.13)	1.37 (0.77, 2.42)
**More than one condition**	1.82 (1.08, 3.07)	1.55 (0.85, 2.83)	1.52 (0.92, 2.50)	1.42 (0.80, 2.51)	1.31 (0.71, 2.40)	1.33 (0.67, 2.63)	1.44 (0.88, 2.36)	1.50 (0.86, 2.64)

Data were analysed using univariable and multivariable logistic regression. * Adjusted for age, BMI, ethnicity, education, income, employment status, marital status, children in the household, age of youngest child. ** Perfect prediction. OR, odds ratio; 95% CI, 95% confidence interval; BMI, body mass index; DM, diabetes mellitus; GDM, gestational diabetes mellitus; GHP, gestational hypertension; PTB, spontaneous preterm birth; SGA infant, small for gestational age; PCOS, polycystic ovary syndrome; CVD, cardiovascular disease; Any pregnancy conditions (including GDM, GHP, pre-eclampsia, PTB, SGA infant); Any non-pregnancy condition (including DM, PCOS, infertility); More than one condition (including DM, GDM, GHP, pre-eclampsia, PTB, SGA infant, PCOS, infertility).

**Table 4:** Association between type 2 diabetes mellitus or cardiovascular disease risk perception and lifestyle behaviours (adjusted and unadjusted).

Lifestyle Behaviours	Type 2 DM (Unadjusted) Coefficient (95% CI)	Type 2 DM (Adjusted *) Coefficient (95% CI)	CVD (Unadjusted) Coefficient (95% CI)	CVD (Adjusted *) Coefficient (95% CI)
**Total physical activity (min/week)**	−56.52 (−374.71, 261.66)	−97.52 (−439.59, 244.56)	−135.03 (−452.54, 182.47)	−261.90 (−605.26, 81.46)
**Total brisk walking (min/week)**	−0.61 (−36.49, 35.26)	−19.11 (−57.76, 19.54)	−13.96 (−49.11, 21.19)	−30.02 (−68.15, 8.11)
**Total moderate activity (min/week)**	−14.53 (−50.57, 21.50)	−29.98 (−66.28, 6.32)	23.57 (−11.67, 58.81)	1.63 (−34.25, 37.50)
**Total vigorous activities (min/week)**	10.48 (−40.55, 61.52)	7.10 (−48.78, 62.97)	8.42 (−43.65, 60.50)	−10.82 (−68.40, 46.75)
**Total sitting time (hours/day)**	0.40 (−0.47, 1.27)	0.66 (−0.33, 1.65)	−0.16 (−1.02, 0.70)	−0.10 (−1.08, 0.88)
**Grain (serve/day)**	0.04 (−0.27, 0.35)	0.22 (−0.11, 0.55)	0.14 (−0.17, 0.45)	**0.41 (0.07, 0.74)**
**Vegetables (serve/day)**	0.22 (−0.08, 0.53)	0.31 (−0.02, 0.65)	0.15 (−0.16, 0.45)	0.08 (−0.26, 0.41)
**Fruit (serve/day)**	−0.10 (−0.32, 0.12)	−0.02 (−0.26, 0.21)	0.17 (−0.06, 0.39)	0.08 (−0.15, 0.32)
**Milk (serve/day)**	0.14 (−0.09, 0.37)	**0.25 (0.01, 0.48)**	0.14 (−0.08, 0.36)	0.19 (−0.05, 0.42)
**Meat (serve/day)**	**0.31 (0.08, 0.53)**	**0.26 (0.01, 0.50)**	0.14 (−0.08, 0.36)	0.09 (−0.15, 0.33)
**Extras (serve/day)**	0.20 (−0.16, 0.57)	0.14 (−0.25, 0.52)	0.16 (−0.20, 0.52)	−0.01 (−0.39, 0.37)

Data analyses using univariable and multivariable logistic regression. * Adjusted for age, BMI, ethnicity, education, income, employment status, marital status, children in the household, and age of youngest child. 95% CI, confidence interval; min/week, minutes per week; serve/day, serve per day; hours/day, hours per day; CVD, cardiovascular disease; DM, diabetes mellitus; Extras, discretionary choices.

## Data Availability

The data presented in this study is not publicly available due to the confidentiality policy of the database, but it is available on resealable request to the corresponding author.

## Supplementary Materials

The following supporting information can be downloaded at: https://www.mdpi.com/article/10.3390/jpm16030137/s1, Table S1: Full report of participant characteristics; Table S2: Subgroup analyses between type 2 diabetes mellitus risk perception and lifestyle behaviours stratified by history of each condition (unadjusted); Table S3: Subgroup analyses between type 2 diabetes mellitus risk perception and lifestyle behaviours stratified by history of each condition (adjusted); Table S4: Subgroup analyses between CVD risk perception and lifestyle behaviours stratified by history of each condition (unadjusted); Table S5: Subgroup analyses between CVD risk perception and lifestyle behaviours stratified by history of each condition (adjusted); Table S6: Subgroup analyses between type 2 diabetes mellitus risk perception and lifestyle behaviours stratified by history of grouped conditions (adjusted and unadjusted); Table S7: Subgroup analyses between CVD risk perception and lifestyle behaviours stratified by history of grouped conditions (adjusted and unadjusted).

## Abbreviations

CVD	Cardiovascular Disease
DM	Diabetes Mellites
GDM	Gestational Diabetes Mellitus
GHP	Gestational Hypertension
PTB	Spontaneous Preterm Birth
SGA infant	Small for Gestational Age Infant
PCOS	Polycystic Ovary Syndrome
BMI	Body Mass Index
SD	Standard Deviation
