# Supplementary material for: Perceived Risk Perception of Future Cardiovascular Disease and Diabetes in the Postpartum Period
Source: J Pers Med. 2026 Mar 1;16(3):137. doi: 10.3390/jpm16030137 (PMC13028156; doi:10.3390/jpm16030137)
Supplement: Supplementary file 1 [file jpm-16-00137-s001.zip › jpm-4090442-supplementary.pdf]

Supplementary

Supplementary Table S1. Full report of participant characteristics

Variable		Number/ Mean (SD or %) n total=497	Any pregnancy conditions		Any non-pregnancy conditions		More than one condition		Overall p-value		
			No n=298 (60%)	Yes n=199 (40%)	No n=424 (85.3%)	Yes n=73 (14.7%)	No n=420 (84.5%)	Yes n=77 (15.5%)	Preg-con	Non-preg-con	More-than
**Age (years)**		33.6 (5.5)	33.5 (5.2)	33.9 (5.9)	33.4 (5.5)	35.1 (5.2)	33.5 (5.4)	34.4 (5.9)	0.357	0.015	0.210
**BMI (kg/m2)**		27.2 (6.9)	26.4 (6.5)	28.4 (7.2)	26.9 (6.5)	29.4 (8.4)	26.7 (6.4)	30.5 (8.3)	0.002	0.004	0.000
**Number of children in house (<18 years old)**		2 (1.1)	1.9 (0.91)	2.2 (1.2)	2 (1)	2.2 (1.2)	1.9 (0.94)	2.5 (1.5)	0.007	0.171	0.000
**Number of adults in house (≥18 years old)**		1.4 (0.84)	1.4 (0.86)	1.4 (0.81)	1.39 (0.86)	1.21 (0.71)	1.37 (0.85)	1.29 (0.78)	0.942	0.088	0.397
**Age of youngest child**		4 (1.9)	4.2 (2.0)	3.8 (1.8)	4 (1.9)	4.5 (1.9)	4.1 (1.9)	3.9 (1.8)	0.049	0.015	0.510
**Country of birth**	Australia	271(54.5)	154 (51.7)	117 (58.8)	217 (51.2)	54 (74)	222 (52.9)	49 (63.6)	0.119	0.000	0.081
	Overseas	226 (45.5)	144 (48.3)	82 (41.2)	207 (48.8)	19 (26)	198 (47.1)	28 (36.4)			
**Marital status**	Never married	30 (6.0)	13 (4.7)	17 (8.5)	26 (6.2)	4 (5.5)	23 (5.5)	7 (9.1)	0.102	0.524	0.274
	Married/De facto	438 (88.1)	269 (90.9)	169 (84.9)	375 (88.9)	63 (86.3)	374 (89.5)	64 (83.1)			
	Separated/ Divorced	27 (5.4)	14 (4.7)	13 (6.5)	21 (5.0)	6 (8.2)	21 (5.0)	6 (7.8)			
**Education level**	Secondary/high school	121 (24.4)	67 (22.7)	54 (27.1)	100 (23.8)	21 (28.8)	98 (23.5)	23 (29.9)	0.112	0.500	0.083
	Diploma/advanced diploma	97 (19.5)	56 (19.0)	41 (20.6)	81 (19.2)	16 (21.9)	79 (18.9)	18 (23.4)			
	University degree	148 (29.8)	84 (28.5)	64 (32.2)	126 (29.9)	22 (30.1)	123 (29.5)	25 (32.5)			
	Graduate/postgraduate degree	128 (25.8)	88 (29.8)	40 (20.1)	114 (27.1)	14 (19.2)	117 (28.1)	11 (14.3)			
**Ethnicity**	Oceanian	257 (51.7)	146 (50.0)	111 (56.6)	208 (49.9)	49 (69.0)	208 (50.4)	49 (65.3)	0.350	0.010	0.052
	Asian	170 (34.2)	108 (37.0)	62 (31.6)	155 (37.2)	15 (21.1)	152 (36.8)	18 (24.0)			
	Other	61 (12.3)	38 (13.0)	23 (11.7)	54 (13.0)	7 (9.9)	53 (12.8)	8 (10.7)			
**Income ($AUD)**	$0-$49,999	76 (15.3)	44 (15.6)	32 (17.4)	64 (16.2)	12 (17.1)	59 (15.1)	17 (23.0)	0.673	0.864	0.198
	$50,000-$99,999	149 (30.0)	90 (31.9)	59 (32.1)	124 (31.3)	25 (35.7)	125 (31.9)	24 (32.4)			
	$100,000-$149,999	148 (29.8)	95 (33.7)	53 (28.8)	128 (32.3)	20 (28.6)	131 (33.4)	17 (23.0)			
	>$150,000	93 (18.7)	53 (18.8)	40 (21.7)	80 (20.2)	13 (18.6)	77 (19.6)	16 (21.6)			
**Employment**	Homemaker/Student/ Government assistance	154 (31.0)	84 (28.7)	70 (35.7)	131 (31.5)	23 (31.5)	122 (29.5)	32 (42.1)	0.137	0.997	0.070
	Full-time employment	149 (30.0)	98 (33.5)	51 (26.0)	127 (30.5)	22 (30.1)	132 (32.0)	17 (22.4)			
	Part-time/Casual employment	186 (37.4)	111 (37.9)	75 (38.3)	158 (38.0)	28 (38.4)	159 (38.5)	27 (35.5)			

Data were analysed according to the type of question asked: normally distributed continues variables (age, BMI) were compared using independent sample t-tests; non-normally distributed continues variables were compared using Mann–Whitney U tests; categorical variables (ethnicity, marital status, education level, employment, conditions) were compared using Pearson's chi-square tests as appropriate. SD, standard deviation; BMI, body mass index; n, number; Any preg-con, Any pregnancy conditions (including GDM, GHP, pre-eclampsia, PTB, SGA infant); Any non-preg-con, Any non-pregnancy condition (including DM, PCOS, infertility); more-than, more than one condition (including GDM, GHP, pre-eclampsia, PTB, SGA infant, DM, PCOS, infertility) ; $AUD, Australian Dollar

Supplementary Table S2. Subgroup analyses between type 2 diabetes mellitus risk perception and lifestyle behaviours stratified by history of each condition (unadjusted)

Lifestyle behaviours	**GDM** CO (95% CI)	**GHP** CO (95% CI)	**PE** CO (95% CI)	**PTB** CO (95% CI)	**SGA infant** CO (95% CI)	**PCOS** CO (95% CI)	**Infertility** CO (95% CI)
Total physical activity (min/week)	-419.03 (-1169.42, 331.36)	**-1096.20 (-2134.34, -58.07)**	-530.25 (-1690.78, 630.28)	-198.39 (-1095.12, 698.35)	-505.09 (-2220.63, 1210.45)	41.89 (-985.95, 1069.73)	464.83 (-843.38, 1773.04)

Total brisk walking (min/week)	-37.20 (-121.81, 47.41)	-98.25 (-215.34, 18.84)	16.99 (-114.22, 148.20)	-53.29 (-154.12, 47.54)	-99.77 (-292.85, 93.31)	-138.37 (-253.53, -23.20)	-52.01 (-199.61, 95.58)
Total moderate activity (min/week)	-6.34 (-91.24, 78.56)	-129.76 (-247.09, -12.42)	11.16 (-120.96, 143.28)	**118.90 (17.89, 219.92)**	-98.89 (-292.73, 94.96)	30.79 (-85.66, 147.24)	3.75 (-144.64, 152.14)
Total vigorous activities (min/week)	-62.23 (-182.61, 58.14)	-166.16 (-332.76, 0.43)	-107.35 (-293.74, 79.03)	-32.81 (-176.59, 110.97)	-76.67 (-351.85, 198.51)	15.85 (-148.86, 180.57)	101.47 (-108.42, 311.35)
Total sitting time (hours/day)	-0.25 (-2.55, 2.05)	1.28 (-1.43, 4.00)	1.62 (-1.37, 4.60)	1.12 (-1.37, 3.61)	**7.42 (1.24, 13.60)**	0.26 (-2.48, 3.00)	1.82 (-1.30, 4.93)
Grain (serve/day)	0.19 (-0.53, 0.91)	-0.11 (-1.11, 0.89)	0.18 (-1.01, 1.36)	-0.50 (-1.37, 0.38)	0.15 (-1.48, 1.78)	0.78 (-0.23, 1.79)	0.22 (-1.05, 1.49)
Vege (serve/day)	-0.28 (-1.01, 0.44)	-0.26 (-1.25, 0.74)	0.43 (-0.71, 1.57)	**-0.86 (-1.71, -0.01)**	-0.83 (-2.43, 0.78)	0.09 (-0.90, 1.09)	0.01 (-1.29, 1.31)
Fruit (serve/day)	-0.01 (-0.53, 0.50)	-0.21 (-0.94, 0.52)	0.07 (-0.79, 0.92)	-0.34 (-0.96, 0.29)	-0.31 (-1.43, 0.82)	0.17 (-0.53, 0.88)	-0.10 (-1.02, 0.83)
Milk (serve/day)	0.28 (-0.25, 0.81)	-0.01 (-0.74, 0.72)	-0.16 (-0.99, 0.67)	**-0.92 (-1.56, -0.29)**	-0.94 (-2.08, 0.21)	-0.17 (-0.91, 0.57)	0.10 (-0.85, 1.04)
Meat (serve/day)	0.19 (-0.33, 0.71)	-0.27 (-1.00, 0.45)	0.47 (-0.38, 1.31)	-0.38 (-1.02, 0.26)	-0.27 (-1.40, 0.87)	0.31 (-0.43, 1.05)	0.25 (-0.68, 1.17)
Extras (serve/day)	0.31 (-0.57, 1.20)	0.33 (-0.85, 1.50)	0.36 (-1.06, 1.77)	-0.87 (-1.92, 0.19)	0.61 (-1.46, 2.67)	-0.00 (-1.21, 1.20)	0.16 (-1.30, 1.62)

Data analysed using univariable logistic regression

CO, coefficient; 95% CI, 95% confidence interval, min/week, minute per week; hours/day, hours per day; GDM, gestational diabetes mellitus; GHP, gestational hypertension; PE, preeclampsia; PTB, spontaneous preterm birth; SGA infant, small for gestational age; PCOS, polycystic ovary syndrome; Extras, discretionary choices

Supplementary Table S3. Subgroup analyses between type 2 diabetes mellitus risk perception and lifestyle behaviours stratified by history of each condition (adjusted)

Lifestyle behaviours	**GDM** CO (95% CI)	**GHP** CO (95% CI)	**Pre-eclampsia** CO (95% CI)	**PTB** CO (95% CI)	**SGA infant** CO (95% CI)	**PCOS** CO (95% CI)	**Infertility** CO (95% CI)
Total physical activity (min/week)	-74.63 (-866.53, 717.28)	-686.80 (-1793.16, 419.55)	-483.64 (-1799.73, 832.45)	-238.02 (-1182.68, 706.63)	-172.72 (-2479.92, 2134.47)	-117.06 (-1189.90, 955.77)	679.28 (-698.78, 2057.34)
Total brisk walking (min/week)	1.44 (-87.95, 90.84)	-71.86 (-196.82, 53.11)	12.53 (-136.34, 161.40)	-54.37 (-160.91, 52.18)	-39.54 (-299.26, 220.17)	**-132.40 (-252.76, -12.03)**	-32.32 (-188.04, 123.40)
Total moderate activity (min/week)	26.11 (-57.69, 109.90)	-92.77 (-209.69, 24.16)	23.81 (-116.42, 164.04)	19.65 (-80.56, 119.85)	-112.77 (-355.79, 130.24)	13.55 (-100.39, 127.51)	5.73 (-140.77, 152.23)
Total vigorous activities (min/week)	-17.38 (-146.72, 111.96)	-121.61 (-302.30, 59.08)	-103.43 (-318.50, 111.64)	-59.73 (-213.94, 94.48)	-54.13 (-430.93, 322.67)	-7.59 (-182.73, 167.54)	156.61 (-68.29, 381.51)
Total sitting time (hours/day)	-0.46 (-3.02, 2.11)	2.59 (-0.62, 5.80)	1.66 (-1.94, 5.27)	1.59 (-1.17, 4.35)	**8.44 (1.85, 15.03)**	0.38 (-2.75, 3.52)	1.71 (-1.84, 5.27)
Grain (serve/day)	0.25 (-0.51, 1.09)	0.03 (−1.04, 1.09)	-0.36 (-1.67, 0.95)	-0.57 (-1.48, 0.34)	1.25 (-0.91, 3.40)	0.66 (-0.40, 1.72)	0.27 (-1.05, 1.59)
Vege (serve/day)	-0.31 (-1.08, 0.46)	0.004 (−1.07, 1.08)	0.04 (-1.23, 1.31)	-0.86 (-1.76, 0.04)	-1.53 (-3.69, 0.63)	-0.07 (-1.13, 0.99)	0.20 (-1.18, 1.57)
Fruit (serve/day)	-0.09 (-0.63, 0.45)	0.26 (−0.51, 1.04)	0.09 (-0.87, 1.04)	-0.35 (-1.00, 0.30)	0.16 (-1.32, 1.64)	-0.12 (-0.85, 0.61)	-0.23 (-1.19, 0.73)
Milk (serve/day)	0.10 (-0.44, 0.65)	0.39 (−0.37, 1.15)	-0.15 (-1.03, 0.74)	-0.62 (-1.28, 0.04)	-0.79 (-2.29, 0.70)	-0.05 (-0.81, 0.71)	0.001 (-0.97, 0.97)

Meat (serve/day)	0.21 (-0.34, 0.76)	0.08 (−0.71, 0.86)	0.54 (-0.39, 1.47)	-0.16 (-0.84, 0.51)	0.39 (-1.12, 1.90)	-0.02 (-0.81, 0.76)	0.02 (-0.95, 0.99)
Extras (serve/day)	0.09 (-0.80, 0.99)	0.24 (−0.96, 1.43)	0.07 (-1.40, 1.54)	-0.65 (-1.71, 0.41)	1.49 (-0.87, 3.84)	0.16 (-1.07, 1.38)	0.04 (-1.42, 1.49)

Data analysed using multivariable logistic regression

Adjusted for age, BMI, ethnicity, education, income, employment status, marital status, children in the household, age of youngest child

CO, coefficient; 95% CI, 95% confidence interval; min/week, minute per week; hours/day, hours per day; GDM, gestational diabetes mellitus; GHP, gestational hypertension; PTB, spontaneous preterm birth; SGA infant, small for gestational age; PCOS, polycystic ovary syndrome; Extras, discretionary choices

Supplementary Table S4. Subgroup analyses between CVD risk perception and lifestyle behaviours stratified by history of each condition (unadjusted)

Lifestyle behaviours	**GDM** CO (95% CI)	**GHP** CO (95% CI)	**Pre-eclampsia** CO (95% CI)	**PTB** CO (95% CI)	**SGA infant** CO (95% CI)	**PCOS** CO (95% CI)	**Infertility** CO (95% CI)	**DM** CO (95% CI)
Total physical activity (min/week)	251.06 (-558.31, 1060.44)	-487.97 (-1547.95, 572.01)	429.7 (-684.8, 1544.2)	-516.81 (-1438.16, 404.54)	-550.26 (-2039.06, 938.53)	418.55 (-649.72, 1486.83)	-599.90 (-1909.64, 709.83)	-276.14 (-1817.92, 1265.63)
Total brisk walking (min/week)	18.58 (-71.03, 108.19)	-76.96 (-194.03, 40.11)	25.8 (-97.8, 149.3)	-74.78 (-176.54, 26.97)	-98.30 (-262.86, 66.27)	-54.28 (-172.42, 63.87)	-12.03 (-157.29, 133.24)	33.88 (-137.01, 204.78)
Total moderate activity (min/week)	19.15 (-70.52, 108.82)	103.06 (-14.21, 220.33)	-19.1 (-143.1, 104.9)	94.45 (-7.60, 196.49)	-116.04 (-280.81, 48.72)	47.17 (-71.53, 165.87)	-37.23 (-182.86, 108.41)	26.30 (-145.03, 197.64)
Total vigorous activities (min/week)	25.81 (-106.90, 158.52)	9.55 (-164.46, 183.56)	57.3 (-125.8, 240.5)	-84.55 (-235.65, 66.55)	-121.35 (-365.41, 122.71)	48.09 (-126.99, 223.17)	-108.97 (-323.92, 105.98)	-37.48 (-289.74, 214.78)
Total sitting time (hours/day)	-1.25 (-3.68, 1.18)	3.84 (1.17, 6.52)	**3.66 (0.71, 6.62)**	0.49 (-1.92, 2.89)	2.70 (-1.41, 6.81)	-0.77 (-3.49, 1.95)	2.18 (-0.94, 5.30)	1.04 (-3.55, 5.64)
Grain (serve/day)	-0.46 (-1.23, 0.31)	0.17 (-0.84, 1.19)	-0.03 (-1.17, 1.10)	-0.15 (-1.04, 0.74)	0.55 (-0.93, 2.04)	0.54 (-0.51, 1.59)	0.57 (-0.68, 1.81)	-0.91 (-2.54, 0.71)
Vege (serve/day)	-0.12 (-0.89, 0.65)	-0.003 (-1.00, 1.00)	0.05 (-1.06, 1.16)	**-1.22 (-2.09, -0.35)**	-0.75 (-2.23, 0.73)	-0.36 (-1.40, 0.68)	-0.02 (-1.26, 1.23)	0.15 (-1.46, 1.77)
Fruit (serve/day)	-0.09 (-0.65, 0.47)	0.69 (-0.05, 1.42)	0.79 (-0.03, 1.61)	-0.13 (-0.78, 0.52)	-0.31 (-1.35, 0.72)	0.41 (-0.33, 1.15)	0.28 (-0.64, 1.20)	0.53 (-0.62, 1.67)
Milk (serve/day)	-0.003 (-0.56, 0.56)	0.34 (-0.39, 1.07)	-0.57 (-1.38, 0.25)	**-0.89 (-1.54, -0.24)**	-0.08 (-1.14, 0.98)	-0.79 (-1.55, -0.03)	-0.56 (-1.50, 0.39)	0.14 (-1.04, 1.31)
Meat (serve/day)	0.34 (-0.22, 0.90)	0.36 (-0.38, 1.10)	-0.17 (-0.99, 0.66)	-0.02 (-0.68, 0.64)	0.31 (-0.74, 1.36)	0.26 (-0.49, 1.01)	0.12 (-0.77, 1.01)	-0.53 (-1.68, 0.61)
Extras (serve/day)	-0.07 (-1.04, 0.90)	0.09 (-1.11, 1.29)	-0.38 (-1.74, 0.98)	-0.63 (-1.75, 0.49)	1.16 (-0.68, 3.01)	-0.37 (-1.57, 0.82)	0.17 (-1.27, 1.62)	-0.94 (-2.91, 1.03)

Data analysed using univariable logistic regression

CO, coefficient; 95% CI, 95% confidence interval; min/week, minute per week; hours/day, hours per day; DM, diabetes mellitus; CVD, cardiovascular disease; GDM, gestational diabetes mellitus; GHP, gestational hypertension; PTB, spontaneous preterm birth; SGA infant, small for gestational age; PCOS, polycystic ovary syndrome; Extras, discretionary choices

Supplementary Table S5. Subgroup analyses between CVD risk perception and lifestyle behaviours stratified by history of each condition (adjusted)

Lifestyle behaviours	**GDM** CO (95% CI)	**GHP** CO (95% CI)	**Pre-eclampsia** CO (95% CI)	**PTB** CO (95% CI)	**SGA infant** CO (95% CI)	**PCOS** CO (95% CI)	**Infertility** CO (95% CI)	**DM** CO (95% CI)
Total physical activity (min/week)	207.78 (-657.03, 1072.58)	-507.29 (-1637.02, 622.44)	902.68 (-347.64, 2153.00)	-596.69 (-1566.82, 373.44)	-146.1 (-1771.96, 1479.66)	84.57 (-1059.36, 1228.50)	-712.34 (-2179.73, 755.05)	-217.85 (-1929.16, 1493.46)

Total brisk walking (min/week)	39.18 (-56.76, 135.13)	-88.92 (-214.13, 36.30)	37.80 (-101.26, 176.87)	-97.76 (-205.23, 9.71)	-100.9 (-280.93, 79.13)	-34.93 (-161.70, 91.84)	15.56 (-147.52, 178.64)	94.24 (-96.37, 284.85)
Total moderate activity (min/week)	17.98 (-72.21, 108.17)	**135.94 (18.75, 253.13)**	-3.30 (-134.39, 127.80)	-2.08 (-103.57, 99.41)	-91.2 (-260.03, 77.59)	22.28 (-97.40, 141.95)	-56.46 (-210.02, 97.09)	53.48 (-125.97, 232.94)
Total vigorous activities (min/week)	45.95 (-98.89, 190.78)	51.45 (-138.13, 241.03)	139.51 (-70.38, 349.40)	-110.81 (-273.42, 51.80)	-84.7 (-356.99, 187.66)	7.07 (-184.58, 198.73)	-112.56 (-358.73, 133.60)	-5.94 (-291.98, 280.10)
Total sitting time (hours/day)	-2.24 (-4.96, 0.47)	**4.80 (1.65, 7.95)**	**3.54 (0.08, 7.00)**	0.74 (-1.93, 3.40)	3.22 (-1.30, 7.75)	-1.09 (-4.33, 2.15)	2.23 (-1.32, 5.79)	2.30 (-2.92, 7.53)
Grain (serve/day)	-0.24 (-1.06, 0.58)	0.22 (-0.86, 1.30)	0.23 (-1.00, 1.46)	-0.01 (-0.94, 0.91)	1.02 (-0.62, 2.65)	0.57 (-0.55, 1.70)	1.02 (-0.34, 2.39)	-0.87 (-2.60, 0.86)
Vege (serve/day)	-0.21 (-1.03, 0.62)	-0.29 (-1.37, 0.79)	-0.18 (-1.41, 1.06)	**-1.20 (-2.12, -0.28)**	-1.51 (-3.17, 0.15)	-0.35 (-1.48, 0.77)	0.17 (-1.21, 1.56)	-0.17 (-1.92, 1.58)
Fruit (serve/day)	-0.25 (-0.84, 0.34)	**0.94 (0.17, 1.71)**	0.77 (-0.13, 1.68)	0.07 (-0.61, 0.74)	0.22 (-0.91, 1.35)	0.27 (-0.51, 1.04)	-0.46 (-1.48, 0.56)	-0.05 (-1.25, 1.15)
Milk (serve/day)	-0.11 (-0.69, 0.47)	0.48 (-0.28, 1.24)	-0.26 (-1.11, 0.60)	-0.46 (-1.13, 0.21)	0.16 (-0.99, 1.31)	-0.71 (-1.50, 0.08)	-0.35 (-1.32, 0.62)	-0.14 (-1.34, 1.06)
Meat (serve/day)	0.13 (-0.47, 0.72)	0.29 (-0.50, 1.08)	-0.11 (-1.01, 0.79)	0.23 (-0.45, 0.92)	0.40 (-0.76, 1.56)	-0.15 (-0.96, 0.65)	0.44 (-0.54, 1.41)	-0.56 (-1.78, 0.66)
Extras (serve/day)	-0.24 (-1.25, 0.78)	-0.27 (-1.49, 0.95)	-0.56 (-1.98, 0.87)	-0.26 (-1.37, 0.85)	0.66 (-1.14, 2.47)	-0.56 (-1.78, 0.66)	0.29 (-1.23, 1.81)	-0.82 (-2.80, 1.16)

Data analysed using multivariable logistic regression

Adjusted for age, BMI, ethnicity, education, income, employment status, marital status, children in the household, age of youngest child

CO, coefficient; 95% CI, 95% confidence interval; min/week, minute per week; hours/day, hours per day; DM, diabetes mellitus; CVD, cardiovascular disease; GDM, gestational diabetes mellitus; GHP, gestational hypertension; PTB, spontaneous preterm birth; SGA infant, small for gestational age; PCOS, polycystic ovary syndrome; Extras, discretionary choices

Supplementary Table S6. Subgroup analyses between type 2 diabetes mellitus risk perception and lifestyle behaviours stratified by history of grouped conditions (adjusted and unadjusted)

Lifestyle behaviours	Any preg-con (Unadjusted) CO (95% CI)	Any preg-con (Adjusted) CO (95% CI)	Any non-preg-con (Unadjusted) CO (95% CI)	Any non-preg-con (Adjusted) CO (95% CI)	More than one-con (Unadjusted) CO (95% CI)	More than-one-con (Adjusted) CO (95% CI)
Total physical activity (min/week)	-578.73 (-1224.37, 66.92)	-267.21 (-944.26, 409.84)	179.88 (-742.85, 1102.61)	156.99 (-808.13, 1122.11)	-390.99 (-1241.82, 459.84)	156.92 (-1051.99, 738.15)
Total brisk walking (min/week)	-54.40 (-127.26, 18.47)	-34.46 (-110.94, 42.01)	-100.51 (-204.15, 3.14)	-90.78 (-199.45, 17.89)	**-100.53 (-196.08, -4.99)**	-68.18 (-168.96, 32.59)
Total moderate activity (min/week)	8.10 (-65.23, 81.43)	-19.34 (-91.17, 52.48)	24.27 (-80.17, 128.72)	20.99 (-81.41, 123.38)	-8.15 (-104.47, 88.17)	15.45 (-79.46, 110.36)
Total vigorous activities (min/week)	-94.60 (-198.15, 8.95)	-60.12 (-170.61, 50.37)	34.85 (-113.10, 182.81)	39.49 (-118.09, 197.07)	-47.46 (-184.00, 89.09)	-22.72 (-168.92, 123.48)
Total sitting time (hours/day)	1.06 (-0.69, 2.82)	1.26 (-0.69, 3.21)	1.06 (-1.32, 3.43)	0.89 (-1.83, 3.61)	2.28 (-0.02, 4.58)	**2.88 (0.21, 5.54)**
Grain (serve/day)	-0.03 (-0.67, 0.60)	0.02 (-0.64, 0.69)	0.45 (-0.46, 1.36)	0.28 (-0.67, 1.23)	0.16 (-0.68, 1.00)	0.01 (-0.87, 0.89)
Vege (serve/day)	-0.39 (-1.01, 0.24)	-0.32 (-0.97, 0.34)	0.07 (-0.84, 0.98)	-0.08 (-1.04, 0.89)	-0.49 (-1.32, 0.34)	-0.57 (-1.45, 0.31)
Fruit (serve/day)	-0.09 (-0.54, 0.36)	-0.02 (-0.48, 0.45)	0.04 (-0.61, 0.69)	-0.16 (-0.83, 0.51)	-0.18 (-0.79, 0.43)	-0.16 (-0.80, 0.47)
Milk (serve/day)	0.23 (0.23, 0.69)	0.29 (-0.17, 0.76)	-0.17 (-0.85, 0.51)	-0.13 (-0.81, 0.56)	-0.48 (-1.09, 0.13)	-0.42 (-1.04, 0.20)
Meat (serve/day)	0.14 (-0.31, 0.59)	0.22 (-0.25, 0.69)	0.35 (-0.33, 1.02)	0.03 (-0.68, 0.74)	0.02 (-0.59, 0.63)	0.18 (-0.46, 0.82)
Extras (serve/day)	0.09 (-0.66, 0.83)	0.02 (-0.73, 0.77)	0.19 (-0.89, 1.27)	0.25 (-0.84, 1.34)	-0.24 (-1.25, 0.77)	-0.38 (-1.39, 0.63)

Data analysed using univariable and multivariable logistic regression

Adjusted for age, BMI, ethnicity, education, income, employment status, marital status, children in the household, age of youngest child

CO, coefficient; 95% CI, 95% confidence interval; min/week, minute per week; hours/day, hours per day; Any preg-con, Any pregnancy conditions (including GDM, GHP, pre-eclampsia, PTB, SGA infant); Any non-preg-con, Any non-pregnancy condition (including, PCOS, infertility); More than-one-con, More than-one condition (including GDM, GHP, pre-eclampsia, PTB, SGA infant, DM, PCOS, infertility); DM, diabetes mellitus

Supplementary Table S7. Subgroup analyses between CVD risk perception and lifestyle behaviours stratified by history of grouped conditions (adjusted and unadjusted)

Lifestyle behaviours	Any preg-con (Unadjusted) CO (95% CI)	Any preg-con (Adjusted) CO (95% CI)	Any non-preg-con (Unadjusted) CO (95% CI)	Any non-preg-con (Adjusted) CO (95% CI)	More than-one-con (Unadjusted) CO (95% CI)	More than-one-con (Adjusted) CO (95% CI)
Total physical activity (min/week)	-321.8 (-980.9, 337.3)	-282.75 (-977.97, 412.46)	252.2 (-594.30, 1098.68)	81.44 (-845.10, 1007.98)	-243.88 (-1056.45, 568.69)	-321.39 (-1202.95, 560.16)
Total brisk walking (min/week)	-47.1 (-121.4, 27.2)	-64.55 (-142.93, 13.83)	-26.73 (-120.45, 66.99)	3.61 (-99.33, 106.56)	-46.75 (-136.56, 43.06)	-31.42 (-129.24, 66.40)
Total moderate activity (min/week)	**77.5 (3.1, 151.9)**	55.37 (-18.67, 129.41)	15.32 (-78.62, 109.26)	2.72 (-94.16, 99.59)	12.10 (-77.92, 102.12)	9.52 (-82.62, 101.65)
Total vigorous activities (min/week)	-29.6 (-135.5, 76.3)	-14.33 (-128.18, 99.52)	33.06 (-105.59, 171.71)	20.48 (-134.63, 175.59)	-51.44 (-184.68, 81.81)	-37.35 (-185.07, 110.38)
Total sitting time (hours/day)	0.84 (-0.93, 2.62)	0.37 (-1.73, 2.46)	0.64 (-1.57, 2.84)	1.11 (-1.49, 3.72)	**3.32 (1.18, 5.47)**	**3.29 (0.82, 5.76)**
Grain (serve/day)	-0.30 (-0.96, 0.35)	-0.06 (-0.74, 0.62)	0.26 (-0.58, 1.10)	0.33 (-0.58, 1.24)	-0.01 (-0.81, 0.79)	0.30 (-0.55, 1.15)
Vege (serve/day)	**-0.70 (-1.33, -0.06)**	**-0.86 (-1.53, -0.19)**	-0.09 (-0.92, 0.75)	-0.11 (-1.04, 0.81)	-0.23 (-1.01, 0.56)	-0.45 (-1.31, 0.40)
Fruit (serve/day)	0.18 (-0.28, 0.64)	0.26 (-0.21, 0.74)	0.43 (-0.18, 1.03)	0.06 (-0.59, 0.71)	0.29 (-0.29, 0.87)	-0.03 (-0.65, 0.58)
Milk (serve/day)	-0.01 (-0.48, 0.45)	-0.01 (-0.49, 0.47)	-0.28 (-0.89, 0.33)	-0.18 (-0.82, 0.45)	-0.58 (-1.16, 0.00)	-0.49 (-1.09, 0.11)
Meat (serve/day)	0.40 (-0.05, 0.86)	0.42 (-0.07, 0.91)	-0.14 (-0.75, 0.46)	-0.24 (-0.90, 0.41)	0.20 (-0.38, 0.78)	0.17 (-0.44, 0.79)
Extras (serve/day)	0.25 (-0.51, 1.01)	0.16 (-0.61, 0.94)	-0.38 (-1.34, 0.58)	-0.19 (-1.19, 0.81)	-0.51 (-1.48, 0.45)	-0.57 (-1.56, 0.42)

Data analysed using univariable and multivariable logistic regression

Adjusted for age, BMI, ethnicity, education, income, employment status, marital status, children in the household, age of youngest child

CO, coefficient; 95% CI, 95% confidence interval; min/week, minute per week; hours/day, hours per day; Any preg-con, Any pregnancy conditions (including GDM, GHP, pre-eclampsia, PTB, SGA infant); Any non-preg-con, Any non-pregnancy condition (including DM, PCOS, infertility); CVD, cardiovascular disease; Extras, discretionary choices
